# The platelet-surface thiol isomerase enzyme ERp57 modulates platelet function

**DOI:** 10.1111/j.1538-7836.2011.04593.x

**Published:** 2012-02

**Authors:** L-M Holbrook, P Sasikumar, R G Stanley, A D SIMMONDS, A B BICKNELL, J M GIBBINS

**Affiliations:** * Institute for Cardiovascular and Metabolic Research, School of Biological Sciences, University of ReadingBerkshire, UK; † Blood transfusion research group, King Saud UniversityRiyadh, Saudi Arabia

**Keywords:** ERp5, ERp57, PDI, platelet activation, redox regulation, thiol isomerase

## Abstract

*Background:* Thiol isomerases are a family of endoplasmic reticulum enzymes which orchestrate redox-based modifications of protein disulphide bonds. Previous studies have identified important roles for the thiol isomerases PDI and ERp5 in the regulation of normal platelet function. *Aim:* Recently, we demonstrated the presence of a further five thiol isomerases at the platelet surface. In this report we aim to report the role of one of these enzymes – ERp57 in the regulation of platelet function. *Methods/Results:* Using enzyme activity function blocking antibodies, we demonstrate a role for ERp57 in platelet aggregation, dense granule secretion, fibrinogen binding, calcium mobilisation and thrombus formation under arterial conditions. In addition to the effects of ERp57 on isolated platelets, we observe the presence of ERp57 in the developing thrombus *in vivo*. Furthermore the inhibition of ERp57 function was found to reduce laser-injury induced arterial thrombus formation in a murine model of thrombosis. *Conclusions:* These data suggest that ERp57 is important for normal platelet function and opens up the possibility that the regulation of platelet function by a range of cell surface thiol isomerases may represent a broad paradigm for the regulation of haemostasis and thrombosis.

## Introduction

Platelets contain a number of thiol isomerase enzymes; PDI [1], ERp5 [2], ERp57, ERp72, ERp44, ERp29 and TMX3 [3]. These are present on the surface membrane of the resting platelet and the levels of which are found to increase during platelet activation [2,4]. Enzyme activity blocking antibodies raised against PDI and ERp5 have been used to demonstrate that the activity of these enzymes contributes to a range of platelet responses including aggregation, adhesion, granule secretion and integrin activation [2,5–7]. Additionally, more recent studies using *in vivo* measurements of thrombus formation show the presence of PDI secreted from platelets and endothelial cells following vascular injury and demonstrate its importance in the thrombus formation process and the activation of coagulation pathways leading to fibrin deposition [8,9]. The discovery of a number of thiol isomerases that are likely to be catalytically competent at the platelet surface is indicative of the existence of an important regulatory paradigm shared by selected thiol isomerases [3]. In this study we investigate the role of the recently identified platelet-surface thiol isomerase, ERp57 in human platelet responses and thrombus formation. ERp57 is a 505 amino acid soluble ER protein [10,11] which is the closest known homologue of PDI, sharing 33% total sequence identity [12,13]. Previous work has attributed important roles for ERp57 in a number of different cell scenarios including; folding of influenza haemagglutinin [14], as a component of MHC peptide loading complexes [15], the modulation of SERCA 2b function in oocytes [16], transcription factor activation [17,18] and the regulation of calcium-mediated capacitation in spermatozoa [19]. In this study, using enzyme activity blocking antibodies, we demonstrate for the first time that cell-surface ERp57 is a key player in the regulation of normal platelet aggregation, integrin activation and signalling. Physiologically, ERp57 is secreted upon vascular injury and accumulates in the thrombus where it regulates the activation and recruitment of other platelets.

## Methods

### Reagents

Cross linked collagen-related peptide (CRP-XL) was purchased from Prof Richard Farndale (University of Cambridge, Cambridge, UK), Protein-G sepharose, cyanogen bromide-activated sepharose and bovine protein disulphide isomerase were from Sigma (Poole, UK). The IV.3 hybridoma cell line (HB-217) was from ATCC (Manassas, VA, USA) and F(ab)′ fragments of purified IV.3 antibody were generated using the Immunopure F(ab)′ purification kit (Pierce, Northumberland, UK). pGEX6P1 expression vector and PreScission protease were from GE Healthcare (Buckinghamshire, UK). Anti-platelet factor 4 antibody was from Accurate Chemical and Scientific Corporation (New York, USA). Anti-human P-selectin phycoerythrin-conjugate was from BD Biosciences (Oxford, UK) and Anti-human fibrinogen FITC-conjugated antibody was from Dako (Cambridgeshire, UK). Anti-GPIb Alexa-488 conjugate was from Emfret Analytics (Germany) Alexa-488 Sheep IgG was from Jackson ImmunoResearch Laboratories (West Grove, Philladelphia, PA, USA). Monoclonal anti-ERp57 (ab13506) and purified mouse IgG was from Abcam (Cambridge, UK). Recombinant human ERp5 was purified as described previously [2] and a construct for the expression of mouse ERp72 was obtained from Dr Mike Green, (St Louis University, USA), DNA was subcloned into pGEX6P1 vector and protein purified as described below for ERp57.

### Antibody preparation

A full length human ERp57 cDNA clone (provided by Prof R Sitia, Instituto Scientifico San Raffaele, Italy) was cloned into the pGEX6P1 expression vector to direct the expression of a soluble ERp57-glutathione *s*-transferase (ERp57-GST) fusion protein in *Escherichia coli.* The fusion protein was purified by affinity chromatography on a glutathione agarose column followed by gel filtration on a Superdex 75 Column (GE Healthcare). ERp57 was cleaved from the GST-fusion partner using PreScission protease following the manufacturer’s protocols (GE Healthcare) and used as an immunogen to raise polyclonal antibodies in sheep. Antibodies were initially purified from serum using protein-G sepharose chromatography and then affinity purified using ERp57 protein immobilised on cyanogen bromide-activated sepharose. Antibodies were eluted from the affinity column as described previously [20] and dialysed against PBS. The ability of affinity purified antibody fractions to inhibit the enzymic activity of recombinant ERp57 was tested by fluorimetric assay based on the reversal of self quenching of the fluorophore dieosin glutathione disulphide (DI-E-GSSG) by reducing agents and enzymes, assayed using a fluorimeter at 525 nm [21]. Antibody cross reactivity assays using recombinant ERp72, PDI and ERp5 were performed in a similar manner. Anti-ERp57 used for *in vivo* experiments was labelled with Alexa-488 using a Microscale labelling kit (Invitrogen, Paisley, UK).

### Platelet preparation and stimulation

Washed human platelets from drug-free donors were prepared by differential centrifugation and suspended to a density of 4 × 10^8^ cells mL^−1^ in Tyrodes-HEPES buffer (134 mm NaCl, 2.9 mm KCl, 0.34 mm Na_2_HPO_4_, 12 mm NaHCO_3_, 20 mm HEPES, 1 mm MgCl_2_ and 5 mm glucose, pH 7.3). The low affinity IgG receptor FcγRIIa, was blocked by pre-incubation with a saturating concentration (6.8 μg mL^−1^) of the F(ab)′ fragment of mAb IV.3 [22] for 1 min and then monoclonal anti-ERp57 (Abcam), anti-ERp57, purified mouse IgG or pre-immune sheep IgG added for 5 min prior to stimulation and briefly stirred. Platelets were stimulated using CRP-XL (1 μg mL^−1^) for 90 s in an optical aggregometer (Chronolog, Havertown, PA, USA) with continuous stirring.

### SDS-PAGE and immunoblotting

Protein separation by reducing SDS–polyacrylamide gel electrophoresis (SDS-PAGE) was performed using 4% stacking and 10% resolving gels. Whole platelet lysate (WPL) or recombinant proteins were transferred to PVDF membrane by means of semi-dry western blotting (Bio-Rad) and membranes blocked using 5% (w/v) BSA in Tris-buffered saline/Tween (TBS-T, 20 mm Tris, 0.14 m NaCl, 0.01% Tween, pH 7.6). Diluted primary antibody (anti-ERp57 1:1000 in 2% (w/v) skimmed milk powder/TBS-T) and species-specific secondary antibody (HRP-conjugated anti-sheep IgG at a dilution of 1:4000) were added for 1 h at room temperature. Blots were washed for 1 h in multiple changes of TBS-T and then visualised using an enhanced chemiluminescence system.

### Platelet granule secretion

ATP secretion from dense granules was measured using lumi-aggregometry. Human platelet rich plasma (PRP) adjusted to 4 × 10^8^ cells mL^−1^ was pre-incubated with 6.8 μg mL^−1^ of the F(ab)′ fragment of the mAb IV.3 (6.8 μg mL^−1^) and anti-ERp57 or pre-immune IgG. Chronolume reagent (50 μL) was added 2 min prior to stimulation with CRP-XL (1 μg mL^−1^). Secretion was recorded for 180 s.

α-Granule secretion was measured by quantifying levels of secreted platelet factor 4 (PF4) by ELISA. Washed human platelets (4 × 10^8^ cells mL^−1^), pre-incubated with anti-ERp57 or pre-immune IgG were stimulated for 90 s using CRP-XL (1 μg mL^−1^) in the presence of EGTA (1 mm), indomethacin (10 μm) and apyrase (2 U mL^−1^). Stimulation was terminated by the addition of 6% (v/v) glutaraldehyde and supernatants obtained by centrifugation. Ten microliters supernatant (1:10 dilution in 0.1 m sodium bicarbonate) was bound to a 96 well plate in triplicate. Following washes with 1 × TBS-T, wells were blocked using 5% (w/v) protease-free BSA. One hundred microliters anti-PF4 antibody (10 ng mL^−1^ in 5% (w/v) BSA) then 100 μL anti-rabbit peroxidise HRP-conjugate (1:1000) were added for an hour. Following washes, the reaction was developed by adding 175 μL 3,3′,5,5′- tetramethyl-benzidine (TMB) substrate then 50 μL 0.5 m hydrochloric acid and absorption read at a wavelength of 450 nm. Alternatively, P-selectin exposure was measured by flow cytometry as reported previously [23].

### Calcium flux measurement *in vitro*

Cytosolic calcium flux (mobilisation from intracellular stores and influx) was measured by spectrofluorimetry [24]. PRP was incubated with Fluo-4NW for 30 min. 6.8 μg mL^−1^ F(ab)′ fragment of mAb IV.3 was added for 1 min, then either anti-ERp57 or pre-immune IgG were added 5 min prior to stimulation. Treated PRP was added to the wells of a reflective-bottomed microtitre plate and calcium flux measured, following stimulation with 1 μg mL^−1^ CRP-XL, using a FLUOstar OPTIMA fluorescence microplate reader (BMG Lab tech, Buckinghamshire, UK).

### Activation of α_IIb_β_3_

The activation state of integrin α_IIb_β_3_ on human platelets was measured by flow cytometry. PRP (4 × 10^8^ cells mL^−1^) treated with 10 μm indomethacin and 2 U mL^−1^apyrase was incubated with 6.8 μg mL^−1^of the mAb IV.3 F(ab)′ fragment for 1 min and then anti-ERp57 or pre-immune IgG for a further 5 min. Following stimulation using CRP-XL (1 μg mL^−1^) for 90 s, platelet-fibrinogen binding was determined by flow cytometry as described previously [23].

### Thrombus formation under arterial flow conditions

Whole human blood was incubated for 20 min with the fluorescent dye DIOC_6_ (1 μm). Blood was perfused over collagen coated capillaries at arterial shear rate (1000 s^−1^) as described by Kulkarni *et al.* [25] in the presence of anti-ERp57 or pre-immune IgG for 3 min. Thrombi were analysed by confocal microscopy. Images from random fields throughout each capillary were captured and Z-stack images obtained at 2 μm intervals which were compiled into a 3D image for the calculation of thrombus volume.

### Platelet static adhesion assay

Human platelet adhesion to collagen and fibrinogen was measured by static adhesion assay and quantified by the measurement of platelet derived acid phosphatase as described previously [26].

### Clot retraction assay

Human PRP (4 × 10^8^ cells mL^−1^) was incubated with either anti-ERp57 or pre-immune IgG. Red blood cells (10 μL) were added to enable visualisation of the developing clot and clot formation initiated by the addition of thrombin (1 U mL^−1^). Clot retraction was allowed to proceed for 2 h around a sealed glass pipette. Clot weights were measured and data normalised to pre-immune IgG controls.

### Measurement of arterial thrombus formation *in vivo*

Intravital microscopy and data analysis were performed as previously described [27]. C57BL/6 mice were anaesthetised by intraperitoneal injection of ketamine (125 mg kg^−1^), xylazine (12.5 mg kg^−1^) and atropine (0.25 mg kg^−1^). Platelets labelled with Alexa-488-conjugated anti-GPIb antibody (0.2 μg g^−1^ weight) were infused through a jugular vein cannula. Following exposure of the testicular cremaster muscle, non-immune IgG or anti-ERp57 (both 2.5 μg g^−1^ mouse weight) were infused and injury upon the cremaster arteriole wall induced using a Micropoint Ablation Laser (Andor Technology, Belfast, UK). The laser injury was optimised to result in the exposure of subendothelial collagen detected through binding of anti-collagen antibodies (data not shown). Thrombi were observed using an upright Olympus BX microscope (Olympus, Essex, UK) and images were captured using a Hamamatsu (Hamamatsu Photonics, Hertfordshire, UK) charge-coupled device camera and data analyzed using Slidebook software Version 5.0 (Intelligent Imaging Innovations, Denver, CO, USA). For the detection of ERp57 at the site of the thrombus, Alexa-488-labelled anti-ERp57 or Alexa-488-labelled sheep non-immune IgG was infused at a concentration of 0.5 μg g^−1^ mouse weight and arterial thrombus formation measured as described above. Animal Experiments were approved by the University of Reading Local Ethical Review Panel and authorized by a UK Home Office Licence.

### Data analysis

Data were analysed using Microsoft Excel and GraphPad Prism software and statistical analysis performed using a paired Student’s *t*-test. For platelet aggregation, dense granule secretion and enzyme activity, pre-immune values in each assay were defined as 100% and values normalised to these values to aid clarity. Non-normalised data was used for all statistical analysis and where possible raw data is displayed. Data where *P* < 0.05 were considered significant.

## Results

### Affinity purified anti-ERp57 inhibits ERp57 enzyme activity

**Fig. 1:**
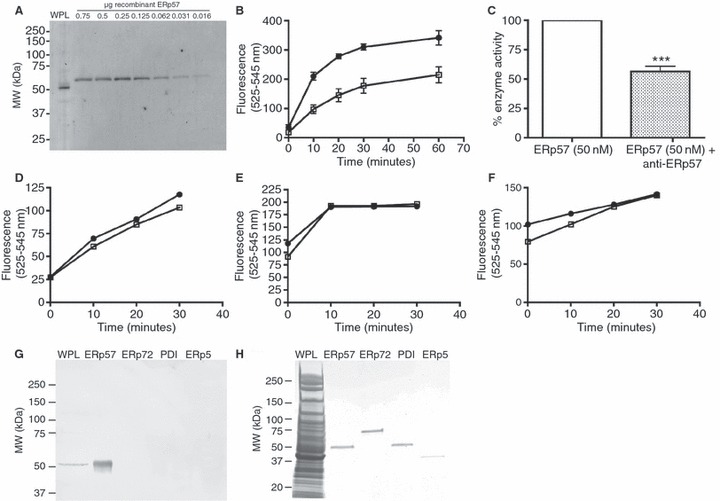
The generation of anti-ERp57 activity blocking antibodies which selectively inhibit ERp57. Human platelets (4 × 10^6^ cells) and recombinant protein (0.75–0.016 μg) were separated by SDS-PAGE and immunoblotted to detect ERp57 (anti-ERp57, 1:1000 dilution in 2% (w/v) skimmed milk powder/TBS-T) and anti-sheep IgG HRP-conjugated antibody (1:4000 dilution in 2% (w/v) skimmed milk powder/TBS-T) then developed using by chemiluminescence (A). The ability of anti-ERp57 (37.5 μg mL^−1^) to inhibit the activity of recombinant ERp57 (50 nm) was measured by fluorescence substrate-based thiol isomerase assay (B). Anti-ERp57 was incubated with enzyme for 5 min prior to addition to 500 μL DI-E-GSSG substrate in the presence of 5 μm DTT (closed circles: enzyme alone, open squares: enzyme with anti-ERp57). Enzyme activity was calculated and % inhibition of ERp57 activity at 30 min demonstrated in graph (C). Cross reactivity of anti-ERp57 with recombinant (D) PDI, (E) ERp72, (F) ERp5 (all 50 nm) was measured by thiol isomerase assay and (G) immunoblotting (5 μg recombinant enzyme/lane). Immunoblots were stained with coomassie blue stain to reveal protein loading (H).

Anti-ERp57 antibodies raised in sheep were purified using immobilised ERp57 protein affinity chromatography in order to select enzyme-activity blocking fractions while preventing cross-reactivity. Antibody sub-populations were analysed by SDS-PAGE and a fluorescence-based enzyme assay using the self-quenching fluorescent substrate Di-E-GSSG was used to determine specificity and establish which antibodies selectively blocked ERp57 activity. As observed previously for ERp5 [2], affinity purification of anti-ERp57 IgG enabled the selection of potent and enzyme-selective inhibitory antibodies which were used to assess the role of ERp57 in platelet regulation. Immunoblots (Fig. 1A) demonstrate that anti-ERp57 recognises a single band in samples of whole platelet lysate (WPL) and recombinant ERp57 and is able to detect ERp57 recombinant protein down to 16 ng mL^−1^, suggesting that anti-ERp57 is both specific and sensitive. Recombinant ERp57 was detected at an apparent higher molecular weight than ERp57 in platelets, due to the presence of a linker attached to the protein that is non-cleavable. Using an assay based on the continuous reduction of the fluorescent substrate DI-E-GSSG over time [21] we observed that recombinant ERp57 (50 nm) has redox activity (Fig. 1B) and that anti-ERp57 (37.5 μg mL^−1^) is able to inhibit this activity by 45% (± 13.8%, Fig. 1B,C). The potential cross reactivity of anti-ERp57 with other closely related platelet thiol isomerase family members was ruled out in the same way using recombinant PDI, ERp72 and ERp5 (Fig. 1D–F respectively). Data from these experiments and also immunoblots of recombinant ERp57, ERp72, ERp5 and PDI proteins probed using anti-ERp57 (Fig. 1G) reveal that anti-ERp57 does not bind to or inhibit the activity of these enzymes.

### ERp57 regulates platelet aggregation and granule secretion

**Fig. 2:**
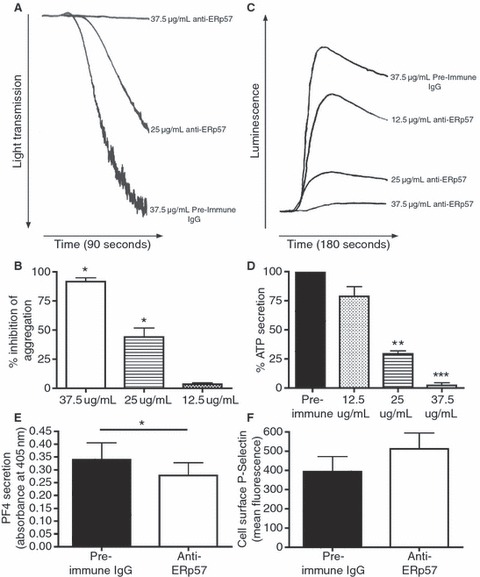
ERp57 regulates human platelet aggregation and granule secretion. Washed human platelets or PRP (4 × 10^8^ cells mL^−1^) were pre-incubated with IV.3 F(ab)′ fragment for 1 min and then either 37.5 μg mL^−1^ pre-immune IgG or anti-ERp57 (12.5–37.5 μg mL^−1^) for 5 min. For aggregometry, platelets were stimulated with 1 μg mL^−1^ CRP-XL and traces recorded for 90 s (A); graph (B) shows % inhibition of aggregation normalised to pre-immune IgG-treated samples. For dense granules, secretion in response to 1 μg mL^−1^ CRP-XL was recorded for 180 s (C) and % ATP secretion normalised to pre-immune IgG-treated samples (D). Platelet factor 4 (PF4) secretion from alpha granules was measured by ELISA. Washed human platelets (4 × 10^8^ cells mL^−1^) were incubated with either pre-immune IgG (37.5 μg mL^−1^) or anti-ERp57 (37.5 μg mL^−1^) for 5 min prior to stimulation (1 μg mL^−1^ CRP-XL). (E) Absorbance values (PF4 levels) from cells stimulated in the presence of anti-ERp57 (open histogram) are presented alongside values obtained for cells incubated with pre-immune IgG (filled histogram), *n* = 5, *P < 0.05. (F) cell surface P-selectin exposure following stimulation with CRP-XL (1 μg mL^−1^) in the presence of either pre-immune IgG (37.5 μg mL^−1^, black graph) or anti-ERp57 (37.5 μg mL^−1^, white graph) was measured by flow cytometry, *n* = 3.

Anti-ERp57 antibodies were used to investigate the importance of ERp57 for human platelet responses. Platelets were stimulated with the collagen-receptor (GPVI)-selective ligand CRP-XL (1 μg mL^−1^) following incubation (5 min) with varying concentrations of anti-ERp57 or pre-immune control IgG. Prior to the addition of inhibitory anti-ERp57, platelets were incubated with a saturating concentration of F(ab)′ fragment of mAb IV.3. to prevent IgG-mediated platelet activation through FcγRIIA [22]. Platelet aggregation in response to CRP-XL was inhibited in a concentration dependant manner following incubation with anti-ERp57 (Fig. 2A,B). At the highest concentration of anti-ERp57 (37.5 μg mL^−1^), aggregation was almost abolished (91% inhibition ± 3.95%). No inhibition of aggregation was observed following incubation of platelets with pre-immune IgG. A control monoclonal anti-ERp57 antibody was used to verify that the effects observed using our affinity purified polyclonal antibody were attributable to enzyme function blocking activity. Human platelets were stimulated with CRP-XL (1 μg mL^−1^) following incubation (5 min) with purified mouse IgG (Fig. S1A), monoclonal anti-ERp57 (Fig. S1B, both 37.5 μg mL^−1^) or the pan thiol isomerase inhibitor bacitracin (3 μg, Fig. S1C). No difference in aggregation between the IgG and mAb treated platelets was observed, while, the positive control bacitracin substantially inhibited platelet aggregation as previously observed [6,28] (Fig. S1D). Thiol isomerase assays using mAb anti-ERp57 (dashed line), mouse IgG (dotted/dashed line) and sheep IgG (dotted line) showed no inhibition of the enzymic activity of ERp57 (Fig. S1E), whereas as shown in Fig. 1B anti-ERp57 (solid line) inhibited ERp57 activity.

To examine whether the observed inhibition of aggregation was due to early disruption of platelet signalling and prevention of the positive feedback effects of granule contents, dense granule secretion was measured. Consistent with the effects on platelet aggregation, pre-incubation with anti-ERp57 inhibited dense granule secretion, with 37.5 μg mL^−1^ anti-ERp57 almost entirely preventing ATP secretion (97% inhibition ± 0.78%) in response to 1 μg mL^−1^ CRP-XL (Fig. 2C,D).

Previous reports have demonstrated that thiol isomerase inhibition diminishes alpha-granule secretion [2,28]. PF4 release was measured by ELISA to determine if ERp57 plays a role in the regulation of alpha granule secretion. PF4 release from activated platelets in the presence of anti-ERp57 (37.5 μg mL^−1^) was found to be reduced modestly (31% ± 19.7% reduction) when compared to the levels observed for pre-immune IgG treated cells (37.5 μg mL^−1^, Fig. 2E). The effect of ERp57 inhibition on P-selectin exposure was determined by flow cytometry, upon stimulation with 1 μg mL^−1^ CRP-XL. Both pre-immune IgG- and anti-ERp57-treated cells displayed a dramatic increase in cell-surface P-selectin levels, with no mean difference (Fig. 2F).

**Fig. 3:**
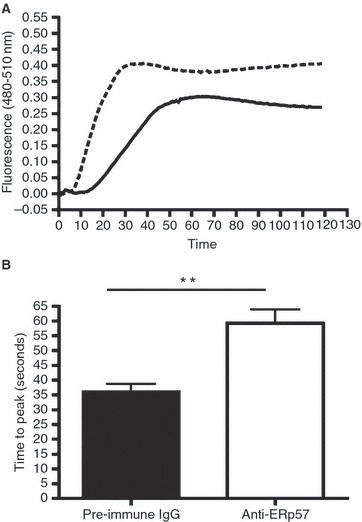
ERp57 regulates calcium flux. Calcium flux *in vitro* was measured by fluorescence based assay. Human PRP, incubated for 30 min with Fluo4NW dye was treated with IV.3 F(ab)′ fragment and then incubated with pre-immune IgG or anti-ERp57 (37.5 μg mL^−1^) for 5 min. Cells (100 μL) were stimulated with 1 μg mL^−1^ CRP-XL and fluorescence measurements taken at 1 s intervals for 120 s. (A) Mean fluorescence measurements for pre-immune IgG treated (dashed line) and anti-ERp57 treated (solid line) cells. The time taken to reach peak fluorescence was measured (B) in the presence of pre-immune IgG (filled) and anti-ERp57 (open). These data are representative of four separate blood donors. *P < 0.05, **P < 0.01, ***P < 0.005.

Given the effects on aggregation and secretion we were interested to determine if early signalling downstream of GPVI was affected by ERp57 inhibition. Calcium flux was measured by fluorimetric assay [24]. In the presence of pre-immune IgG (37.5 μg mL^−1^) calcium flux was rapid with a mean time to peak fluorescence of 36 s (± 3.16 s) whereas anti-ERp57 (37.5 μg mL^−1^) delayed the time to peak to 59 s (± 5.44 s, Fig. 3A,B). Peak fluorescence levels were also reduced by 53.7% in the presence of anti-ERp57, compared with pre-immune IgG treated cells (data not shown). Previous reports using anti-ERp5 inhibitory antibodies, showed that ERp5 inhibition did not impact on calcium mobilisation [2], whereas ERp57 inhibition markedly decreases and delays cytosolic calcium elevation possibly through inhibition of mobilisation or influx. These data together with the alpha granule data suggest that within the thiol isomerase family, different family members interact with distinct cell-surface proteins and/or signalling pathways with potentially differing modes of action.

### Fibrinogen binding is inhibited by ERp57

**Fig. 4:**
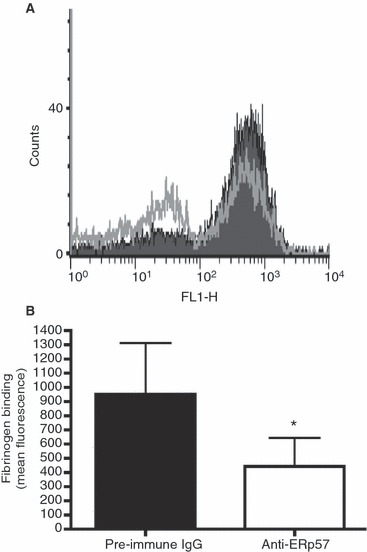
ERp57 inhibition reduces platelet fibrinogen binding. Fibrinogen binding to integrin α_IIb_β_3_ in the presence of either pre-immune IgG (37.5 μg mL^−1^, filled histogram) or anti-ERp57 (37.5 μg mL^−1^, open histogram was measured by flow cytometry. Human platelets were stimulated with CRP-XL (1 μg mL^−1^) and ligated fibrinogen detected using FITC-labelled anti-fibrinogen antibody (A). Fluorescence data for pre-immune IgG and anti-ERp57 treated cells from 3 platelet donors (B), *P < 0.05.

Since α-granule secretion and therefore fibrinogen secretion is less sensitive to the inhibition of ERp57, we investigated whether the inhibition of aggregation was due to an impairment of α_IIb_β_3_ activation. Indeed, inhibition of ERp5 or PDI has been shown to exert an inhibitory effect on this receptor, preventing activation and fibrinogen ligation [2,5]. The levels of fibrinogen binding at the surface of human platelets was assessed by flow cytometry. ERp57 inhibition resulted in a typical two-peaked profile, representing two populations of platelets – activated and non-activated (Fig. 4A). Compared with the pre-immune IgG treated platelets, anti-ERp57 pre-incubation was found to decrease CRP-XL stimulated binding of fibrinogen to platelets by 67% (± 6.0%, Fig. 4B).

### ERp57 inhibition effects adhesion and integrin mediated signalling

**Fig. 5:**
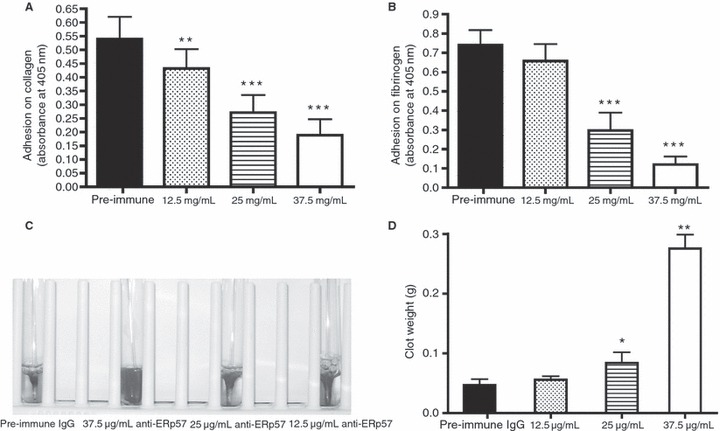
ERp57 is involved in integrin mediated adhesion and clot retraction. Microtitre plates were coated with (A) collagen (20 μg mL^−1^) or (B) fibrinogen (1 mg mL^−1^). Platelets pre-treated with F(ab)′ fragment of IV.3 antibody and then incubated with either pre-immune IgG (37.5 μg mL^−1^) or anti-ERp57 (12.5–37.5 μg mL^−1^) were allowed to adhere. Adherent cells were lysed with acidic lysis buffer and the assay developed using *p*-NPP substrate. Absorbance data represent 4 separate experiments, **P < 0.01, ***P < 0.005. Clot retraction was measured using human PRP adjusted to 4 × 10^8^ cells mL^−1^. Cells were incubated with either pre-immune IgG (37.5 μg mL^−1^) or anti-ERp57 (12.5–37.5 μg mL^−1^) for 5 min. (C) Clots formed in the presence of varying concentrations of anti-ERp57 after 2 h and (D) mean data for clot weight. *n* = 4, *P < 0.05, **P < 0.01.

Previous reports have demonstrated that thiol isomerase activity is important for integrin-mediated adhesion [2,5]. Adhesion to collagen and fibrinogen coated surfaces was measured by static adhesion assay as described previously [26]. Platelet adhesion following pre-incubation with anti-ERp57 (12.5, 25 or 37.5 μg mL^−1^) was inhibited in a concentration-dependant manner on both matrices (Fig. 5A,B). At the highest concentration examined (37.5 μg mL^−1^), anti-ERp57 inhibited adhesion onto collagen by 65% (± 26.5%) and onto fibrinogen by 83.7% (± 34%).

In addition to the effects on initial platelet signalling and later events we observed that during the antibody generation process, immunisation with recombinant ERp57 caused a progressive defect in clot formation following blood sampling from the sheep. To investigate whether this defect was due to an effect on outside in signalling through integrin α_IIb_β_3_, clot retraction assays were performed [29]. Figure 5C shows representative clots which were retracted over a period of 2 h in the presence of either pre-immune IgG or varying concentrations of anti-ERp57. Analysis of the weight of each clot revealed that clots formed in the presence of pre-immune IgG (37.5 μg mL^−1^) were fully retracted with a mean weight of 0.047 g (± 0.011 g) whereas the presence of anti-ERp57 caused inhibition of clot retraction with clots weights as follows: 0.275 ± 0.027 g (37.5 μg mL^−1^), 0.084 ± 0.020 g (25 μg mL^−1^) and 0.055 ± 0.0068 g (12.5 μg mL^−1^, anti-ERp57, Fig. 5D). These data are consistent with the ability of ERp57 to modulate both inside-out platelet integrin signalling, and the outside-in integrin signalling that accompanies platelet activation.

### ERp57 effects thrombus formation

**Fig. 6:**
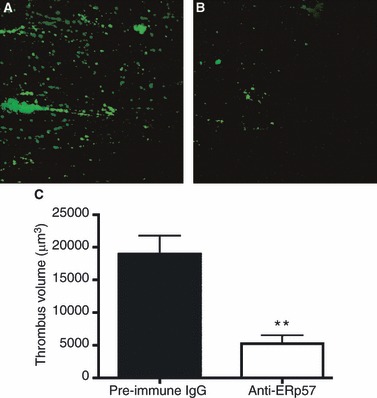
ERp57 modulates *in vitro* thrombus formation. Human whole blood labelled with DIOC_6_ was incubated with either (A) pre-immune IgG, 37.5 μg mL^−1^ or (B) anti-ERp57, 37.5 μg mL^−1^ for 5 min and then perfused over collagen coated capillaries at a shear rate of 1000 s^−1^ for 3 min. Randomly selected fields were visualised and quantified by confocal microscopy (images A and B) and thrombus volume measurements obtained (C), *n* = 3, **P < 0.01.

In order to assess the potential physiological significance of ERp57, an *in vitro* thrombus formation model was used. Whole human blood labelled with DIOC_6_ was perfused through collagen coated glass capillaries at an arterial shear rate of 1000 s^−1^ in the presence of either pre-immune IgG or anti-ERp57 (both 37.5 μg mL^−1^). Adherent thrombus volume was quantified by confocal microscopy. Capillaries from pre-immune IgG treated blood showed widespread coverage with thrombi (Fig. 6A) whereas the capillaries from anti-ERp57 treated blood showed sparse thrombus coverage (Fig. 6B). Analysis of the volume of thrombi in multiple capillaries revealed that ERp57 inhibition resulted in a 78% decrease in thrombus volume compared to pre-immune IgG-treated blood (Fig. 6C). These data are consistent with the observed effects on platelet aggregation and adhesion onto collagen suggesting that ERp57 plays an important role in both initial adhesion and the formation of stable aggregates under arterial flow conditions.

### ERp57 is released at sites of vascular injury and regulates arterial thrombus formation

**Fig. 7:**
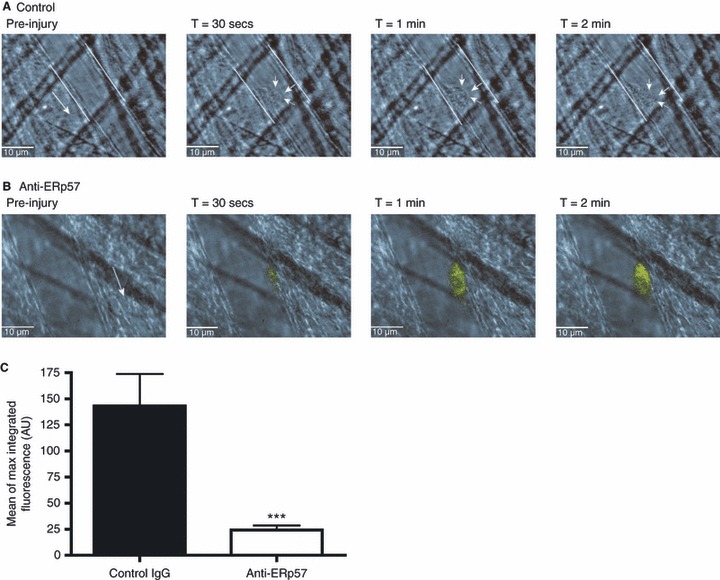
ERp57 modulates arterial thrombus formation *in vivo.* The presence of ERp57 in thrombi and its role in arterial thrombus formation was examined by intravital microscopy using a laser injury model. Alexa-488 labelled control-IgG (A) or anti-ERp57 (B) were infused into C57/BL6 mice, and following laser injury images were captured over 2 min of thrombus formation (*n* = 3). (C) The effect of anti-ERp57 on arterial thrombus formation was quantified following laser injury in mice treated with Alexa-488 anti-GPIb-conjugated antibody. Arrows denote the site of the thrombus. Peak integrated thrombus fluorescence measurements for thrombi formed in the presence of anti-ERp57 (open histogram) treated mice were compared to those obtained for control IgG (closed histogram). *n* = 24, ***P < 0.005.

Having established that ERp57 plays an important role in the regulation of platelet aggregation and integrin mediated events, we sought to determine if extracellular ERp57 plays a role in the regulation of arterial thrombus formation *in vivo*. This was examined using a laser-induced model of thrombosis in mice and visualised by intravital microscopy. Following infusion of Alexa-488 labelled anti-ERp57 antibodies, time dependant localisation of fluorescence within the platelet thrombus was observed (Fig. 7B), consistent with the binding of ERp57 to the surface of platelets [3].

The importance of ERp57 in arterial thrombus formation was determined using inhibitory and unlabelled anti-ERp57 and platelets labelled with anti-GPIb Alexa-488 conjugate. Following laser-induced injury, integrated fluorescence of the thrombi formed, and therefore platelet accumulation, was measured. Compared to the control IgG treated mice, thrombus formation in the presence of anti-ERp57 was reduced substantially (83% reduction in mean peak thrombus fluorescence levels) (Fig. 7C). These data are consistent with our hypothesis that ERp57 located on the platelet surface is important for arterial thrombus formation.

## Discussion

Platelets contain multiple thiol isomerases which are released and bind the cell surface [1–3,28]. One of these enzymes, ERp57, is known to play diverse roles in other cell types but until recently little was known of its role in platelet function. In this study we demonstrate an important role for ERp57 in multiple components of platelet function and arterial thrombus formation.

ERp57 is a component of protein folding complexes in the ER, it is involved in fertilisation and forms complexes with gene transcription factors, making its genetic ablation embryonically lethal. Genetic knockdown approaches have been deployed to study the role of ERp57 but developmental adaptive effects have complicated assessment of its function [30]. Other approaches to thiol isomerase inhibition include the use of the non-specific inhibitor bacitracin, thiol blocking reagents or enzyme function blocking antibodies which thus far have proved the most successful and allow for the targeting of individual enzymes. Indeed these reagents have been key to developing an understanding of the roles of PDI and ERp5 in platelet function. Despite the availability of an antibody that is reported to block ERp57 activity on the surface of spermatozoa [19], experiments using this antibody, showed no effect on either platelet function or thiol isomerase activity (Fig. S1), and therefore prior to this study, no reagents which selectively block ERp57 activity at the platelet surface were available. We have developed a function blocking antibody that allows for the acute and selective inhibition of ERp57 function and for the first time have demonstrated the significance of cell surface ERp57 in platelet function.

Incubation of anti-ERp57 with human platelets *in vitro* resulted in inhibition of both platelet aggregation and dense granule secretion following stimulation with the GPVI agonist CRP-XL. Pre-immune IgG and monoclonal anti-ERp57 did not inhibit ERp57 enzymic activity or platelet responses to agonist. The levels of inhibition are greater than observed in previous reports in which both PDI and ERp5 were inhibited using function blocking antibodies to both enzymes but where complete inhibition was not observed. It is therefore possible that ERp57 plays an important role normal platelet function. ERp57 inhibition was found to have a distinct effect on calcium flux, causing a decrease in the levels and rate of change of cytosolic calcium, whereas the inhibition of another closely related enzyme, ERp5, has little effect on calcium mobilisation [2].

Furthermore, in contrast with previous reports that show both ERp5 and PDI regulate α-granule secretion, in this report we determined using two different assays that α granule secretion is less sensitive to the effects of ERp57 inhibition, whereas dense granule secretion is inhibited substantially. Since α-granule and therefore fibrinogen secretion were normal in the presence of anti-ERp57, we investigated fibrinogen binding and outside-in signalling following fibrinogen ligation and found that these were both impaired by ERp57 inhibition. It is therefore possible that ERp57 at the cell surface may be involved in the regulation of the redox changes associated with activation of integrin α_IIb_β_3_ [31] leading to an enhancement of fibrinogen ligation and associated outside-in signalling. Taken together these data are indicative of some fundamental differences in the roles played by the different cell-surface thiol isomerases in the regulation of platelet activation. The mechanisms that underlie the ability of extracellular ERp57 to modulate dense granule secretion to a greater extent than α-granule secretion will be of particular interest for future characterisation.

Platelet adhesion on to collagen and fibrinogen coated surfaces was found to be reduced through the inhibition of ERp57 in the absence of an activatory stimulus. We believe that cell-surface ERp57 may be in part responsible for the modulation of tonic signalling levels and that perturbations of this lead to the observed reduction in initial adhesion which is also likely to account for the decreased formation of thrombi formed under *in vitro* flow conditions.

Using an *in vivo* approach we have determined that like PDI [8], ERp57 is secreted at the site of vascular injury, where it begins to accumulate within the platelet thrombus. Inhibitory anti-ERp57 was found to decrease arterial thrombosis suggesting that extracellular ERp57 within a developing platelet thrombus acts as a positive regulator of the thrombus formation process. Furthermore, ERp57 release following GPVI stimulation of human platelets, has been reported to support the activation of tissue factor leading to increased activation of coagulation [32]. The inhibition of ERp57 at sites of arterial thrombosis may therefore also impact on the regulation of coagulation along with other released thiol isomerases. The ERp57 recruited to platelet thrombi may be derived from platelets themselves, as we have shown *in vitro* in isolated platelet preparations [3], but additional sources such as laser-damaged/activated endothelium may also contribute. Indeed human umbilical vein endothelial cells have been shown to express both ERp57 [3] and PDI, and laser injury has been shown to cause PDI release from endothelial cells [8].

Based on the data presented in this report, we propose that ERp57 plays an important role in both the regulation of the initial events leading to platelet activation and the later stages which support arterial thrombus formation. ERp57 shares some mechanistic overlap with the other platelet thiol isomerases ERp5 and PDI, but emerging data indicate distinctive substrate specificities, binding partners and mechanisms of action by different members of the platelet thiol isomerase family at the platelet surface. While further understanding of the specific substrates of the individual thiol isomerases is required, our working hypothesis is that ERp57 becomes enriched on the surface of activated platelets and serves to enhance or stabilise the transition in integrin affinity from an inactive state to a ligand binding conformer by catalysing the remodelling of disulphide bonds within the receptor itself or other, as of yet unidentified substrates. This results in enhanced platelet adhesion, aggregation, outside-in signalling, and therefore activation and thrombus formation levels. We recently reported the identification of a further four platelet-surface thiol isomerase enzymes, ERp72, ERp44, ERp29 and TMX3 [3] which together with ERp57, ERp5 and PDI may contribute to a paradigm for platelet regulation that is of physiological importance for haemostasis and thrombosis.

## Supporting Information

**Figure S1.** Monoclonal anti-ERp57 and control IgG do not inhibit platelet reactivity or ERp57 enzyme activity.
