# Supplementary figures and images for: The platelet-surface thiol isomerase enzyme ERp57 modulates platelet function
Source: J Thromb Haemost. 2012 Feb;10(2):278–88. doi: 10.1111/j.1538-7836.2011.04593.x (PMC3444690; doi:10.1111/j.1538-7836.2011.04593.x)

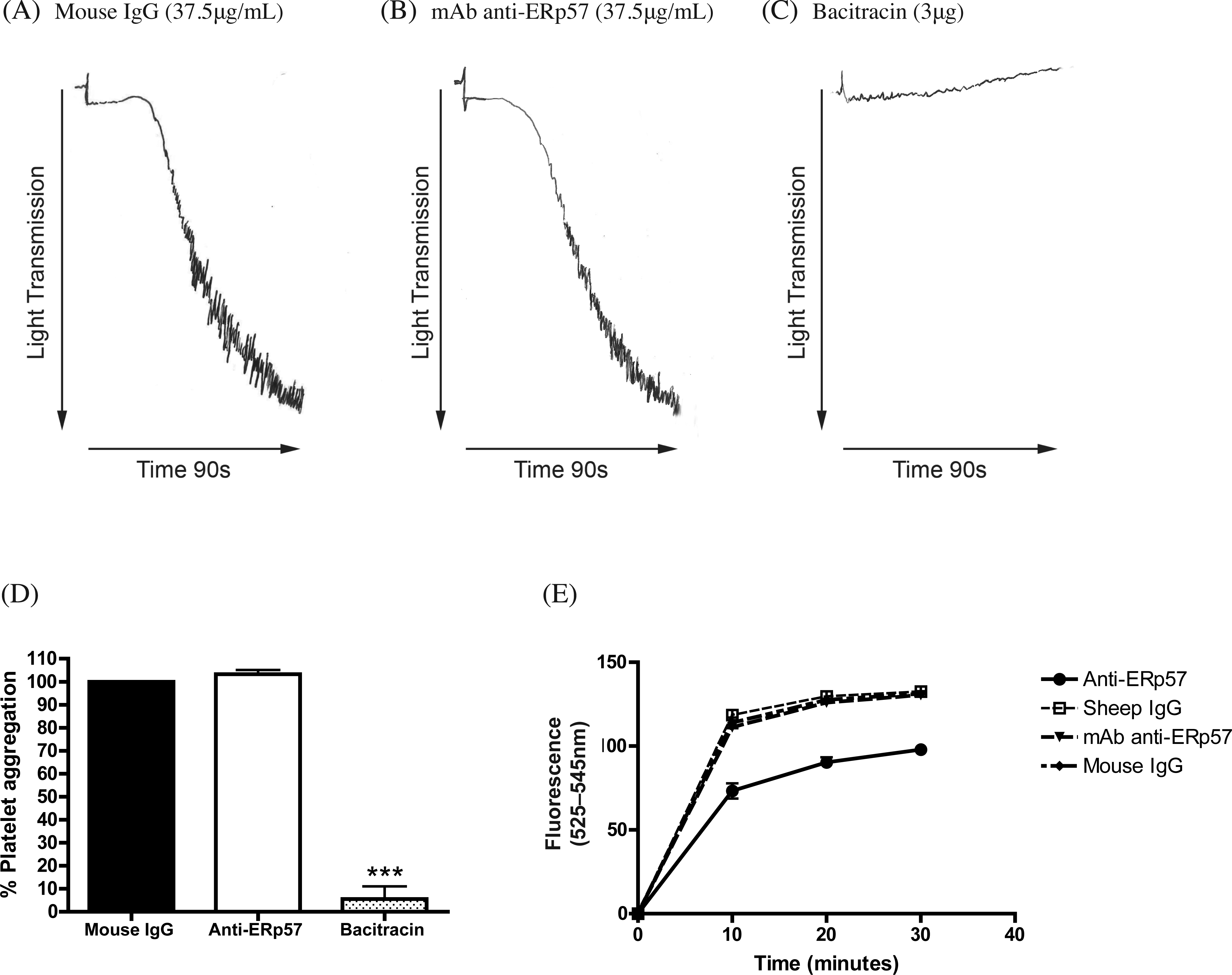

Supplement: Supplementary file 1 [file jth0010-0278-SD1.tif]
